# Glycol Chitosan: A Water-Soluble Polymer for Cell Imaging and Drug Delivery

**DOI:** 10.3390/molecules24234371

**Published:** 2019-11-29

**Authors:** Fengming Lin, Hao-Ran Jia, Fu-Gen Wu

**Affiliations:** State Key Laboratory of Bioelectronics, School of Biological Science and Medical Engineering, Southeast University, Nanjing 210096, China; linfengming@seu.edu.cn (F.L.); jiahr@seu.edu.cn (H.-R.J.)

**Keywords:** polymeric nanoparticles, fluorescence imaging, antibacterial, anticancer, supramolecular self-assembly

## Abstract

Glycol chitosan (GC), a water-soluble chitosan derivative with hydrophilic ethylene glycol branches, has both hydrophobic segments for the encapsulation of various drugs and reactive functional groups for facile chemical modifications. Over the past two decades, a variety of molecules have been physically encapsulated within or chemically conjugated with GC and its derivatives to construct a wide range of functional biomaterials. This review summarizes the recent advances of GC-based materials in cell surface labeling, multimodal tumor imaging, and encapsulation and delivery of drugs (including chemotherapeutics, photosensitizers, nucleic acids, and antimicrobial agents) for combating cancers and microbial infections. Besides, different strategies for GC modifications are also highlighted with the aim to shed light on how to endow GC and its derivatives with desirable properties for therapeutic purposes. In addition, we discuss both the promises and challenges of the GC-derived biomaterials.

## 1. Introduction

Both cancer and microbial infections are major threats to human health and have become the leading causes of death in the world for decades [1,2]. To date, a variety of systemically delivered drug carriers such as inorganic nanovehicles, liposomes, and polymeric nanoparticles (NPs) have been developed to ferry different types of drugs including small molecules, nucleic acids, and peptides/proteins, with the aim to enhance their therapeutic efficacies against tumors and microbial infections with better biosafety [3]. Among them, polysaccharides have been widely demonstrated as a class of excellent drug carriers because of their good biocompatibility/biodegradability and low cost [4]. Chitosan is a linear polysaccharide derived by the deacetylation of the *N*-acetyl glucosamine units of chitin, a natural polymer from crustacean shells, through hydrolysis at high temperatures under alkaline conditions. Chitosan has been widely applied in the biomedical field, for it is biocompatible, biodegradable, and abundant in nature. However, chitosan is typically insoluble in water above pH 6 and requires the addition of acid to ensure the protonation of the primary amines, which greatly limits its further modifications for desired purposes. Glycol chitosan (GC) is a chitosan derivative conjugated with hydrophilic ethylene glycol branches that render the polymer soluble in water at a neutral/acidic pH. The molecular weight of GC ranges from 20 to 250 kDa with the degree of deacetylation from 60 to 82.7% [5]. The reactive functional groups of GC such as amine and hydroxyl groups provide flexibility for various chemical modifications to form a number of derivatives. These GC derivatives form self-assembled nanostructures or hydrogels, serving as excellent drug delivery systems for diagnostic agents and therapeutic drugs to combat cancers and pathogenic microorganisms.

**Figure 1:**
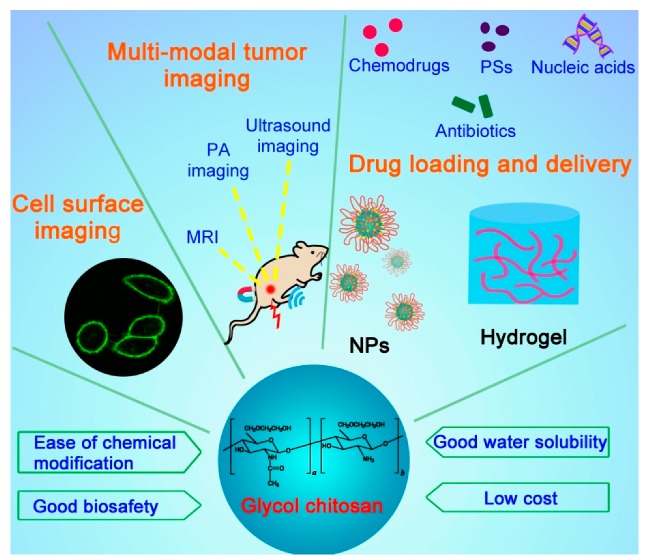
Schematic illustrating the structure, properties, and applications of glycol chitosan.

This review describes the recent progress of GC-based materials in cell imaging and drug delivery applications for diagnosing and treating different diseases, especially cancer and pathogen infections (Figure 1). We also summarize the smart drug delivery strategies on the basis of GC for passive and active tumor-targeted drug delivery, stimuli-responsive drug release, and combination therapy.

## 2. GC Derivatives for Imaging Applications

### 2.1. Cell Surface Imaging

**Figure 2:**
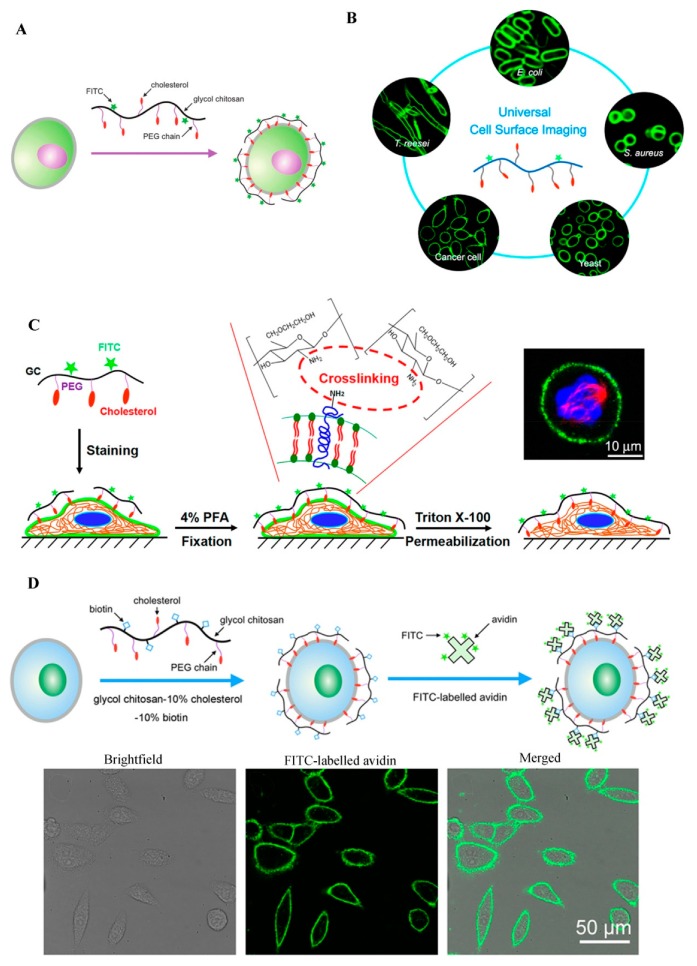
GC-based cell surface imaging. (**A**) Schematic displaying the plasma membrane labeling of Chito-Chol-FITC. Reproduced with permission from Ref. [6]. Copyright 2015 Royal Society of Chemistry. (**B**) Schematic illustration of the universal cell surface imaging of animal cells, bacteria, and fungi using Chito-Chol-FITC. Reproduced with permission from Ref. [7]. Copyright 2016 American Chemical Society. (**C**) Mechanistic diagram of the anti-permeabilization property of Chito-Chol-FITC during immunofluorescence staining. Reproduced with permission from Ref. [8]. Copyright 2017 American Chemical Society. (**D**) Schematic illustration of long-time cell membrane labeling using a two-step modification method through the recognition of FITC-labeled avidin with biotin. Reproduced with permission from Ref. [9]. Copyright 2016 American Chemical Society.

The plasma membranes of cells are involved in many biological events such as signal transduction, endocytosis, exocytosis, cell migration, cell adhesion, cell proliferation, and apoptosis [6]. Cell surface labeling is a powerful tool to study these plasma membrane-related cellular behaviors. Typically, plasma membrane dyes are small molecules, which can only label the cell surface for a short time period due to their fast cellular internalization characteristic [6]. Therefore, it is necessary to develop a fluorescent dye for stable plasma membrane imaging. Recently, our group has developed a GC-based fluorescent probe by linking GC with cholesterol-polyethylene glycol (PEG-Chol) and fluorescein isothiocyanate (FITC), and successfully applied this probe (termed Chito-Chol-FITC, GC-PEG cholesterol-FITC, or GC-PEG Chol-FITC) to cell surface labeling [6,7,8]. The Chito-Chol-FITC could bind to the plasma membranes of mammalian cells through the insertion of the hydrophobic cholesterol units into the lipid bilayers (Figure 2A). The imaging results revealed that Chito-Chol-FITC rapidly stained the cell membrane within 5 min and resisted cellular internalization for up to 6 h [6]. What is more, Chito-Chol-FITC realized the universal imaging of the plasma membranes of mammalian cells (by hydrophobic interaction) and the cell walls of fungal and bacterial cells (by electrostatic interaction) (Figure 2B) [7]. Notably, Chito-Chol-FITC did not detach from the cell surface after permeabilization treatment during immunofluorescence staining and was compatible with the immunofluorescence staining for the simultaneous labeling of the plasma membrane and cytoskeletons (Figure 2C), ensuring the clear observation of binucleated cells and metaphase cells [8]. The anti-permeabilization property of Chito-Chol-FITC was attributed to its large molecular weight and the amine crosslinking between Chito-Chol-FITC and the membrane proteins/lipids induced by the addition of paraformaldehyde in the fixation step. The imaging performance of Chito-Chol-FITC far surpassed that of the commercial plasma membrane dyes like FM families and DiD, which were internalized by the cells in 10–15 min. In addition to the facile synthesis and low cost of Chito-Chol-FITC, this probe is promising to serve as a novel and valuable platform for studying cell surface-related biological events and advancing cell surface engineering. In another work by the same group, long-time plasma membrane imaging was realized by the supramolecular recognition between GC-10% PEG2000 cholesterol-10% biotin (GC-Chol-Biotin) and FITC-conjugated avidin (avidin-FITC) (Figure 2D) [9]. This two-step strategy presented a stable plasma membrane imaging for up to 8 h without substantial internalization or detachment of the dyes, and outperformed the current commercial plasma membrane imaging agents such as CellMask and DiD. Furthermore, the authors used this imaging method to dynamically monitor different plasma membrane behaviors including plasma membrane vesiculation, membrane blebbing, and cell shrinkage. Besides being used to image the whole plasma membranes, GC was also designed to visualize specific membrane structures, e.g., lipid raft domains, by grafting GC with FITC [10]. In summary, GC polymers can serve as a promising platform for constructing various cell surface probes with desirable properties, benefiting from their good water solubility, ease of chemical modification, and low cytotoxicity.

### 2.2. GC Derivatives for In Vivo Cancer Diagnosis 

Detecting cancers at the early stages before their metastasis occurs through the lymph systems and blood vessels is very important for cancer treatments, but still remains challenging. To date, different noninvasive imaging modalities like X-ray, computed tomography (CT), magnetic resonance imaging (MRI), ultrasound (US), positron emission tomography (PET), optical fluorescence imaging, and single photon emission computed tomography (SPECT) have been employed for the detection of cancers in the early stages [11]. These imaging strategies are also critical for the discovery and development of novel drugs by monitoring drug responses and distributions in real time and measuring biological changes in living systems. Recently, GC and its derivatives have been commonly used to modify or load various imaging agents, e.g., iron oxide NPs [12,13] and gadolinium [14], for improving their cancer diagnostic outcomes. In 2011, Yuk et al. prepared a GC/heparin-immobilized iron oxide NPs (composite NPs) to achieve MRI [12]. Since both iron oxide NPs and GC NPs are cationic, gold was deposited on the surface of the cationic iron oxide NPs to introduce negative charges on the surface, ensuring the electrostatic interaction between the iron oxide NPs and GC NPs in the aqueous media. Gold-deposited iron oxide NPs were immobilized into the GC/heparin network to form the composite NPs. The composite NPs were stabilized with heparin via the electronic interaction between cationic GC and anionic heparin. The composite NPs showed improved T_2_* negative images as compared with Resovist, and realized highly selective tumor imaging.

Meanwhile, GC has been used to coat gold NPs (GC-AuNPs) as an imaging contrast agent for the US-guided photoacoustic (USPA) imaging of sentinel lymph node (SLN) metastases [15]. The USPA immunofunctional imaging successfully discriminated metastatic lymph nodes from non-metastatic ones [15], which can help physicians to detect micrometastases by SLN biopsy. GC-AuNPs were conjugated with 4-azidobenzoic acid (AzBA), yielding AzGC-AuNPs [16]. AzBA groups produced nitrogen gas (N_2_) through photolysis to form echogenic N_2_ microbubbles for the enhanced contrast in US imaging. Owing to their small size (less than 100 nm), AzGC-AuNPs could penetrate the endothelial barrier for diagnosis of diseases, which outperformed conventional microbubbles. Together with the generated negative zeta potentials after gas generation, this small size of AzGC-AuNPs brought about excellent blood residency and clearance. Therefore, AzGC-AuNPs have great potential to serve as a US contrast agent for US imaging and other contrast-enhanced imaging methods such as MRI and optical coherence tomography. One limitation of AzGC-AuNPs is its optical absorption at low wavelengths (i.e., 520–530 nm), which can be solved by using gold nanorods or other particles with absorption in the near-infrared (NIR) region.

Multimodal imaging can provide more credible and accurate imaging of focal areas by overcoming the limitations of single-modal imaging. MRI is one of the most powerful diagnostic approaches for three-dimensional human body imaging with high spatial resolution and deep tissue penetration. However, MRI shows low sensitivity to contrast agents, requiring a long time and a high dose of the agent for effective imaging. By contrast, optical imaging with NIR fluorescence (NIRF) allows a rapid screening of diseases with a low dose, but has poor tissue penetration. Consequently, the dual-modal imaging via NIRF imaging and MRI is believed to overcome the intrinsic disadvantages of single-modal NIRF imaging and single-modal MRI. For example, Nam and co-workers developed an NIRF and MRI dual-modality imaging agent using GC NPs, an effective positive MRI contrast agent (gadolinium), and NIRF dye (cyanine 5.5, Cy5.5) [14]. 1,4,7,10-Tetraazacyclododecane-1,4,7,10-tetraacetic acid (DOTA) for gadolinium chelating was conjugated to GC-5β-cholanic acid (GC-CA) to generate Cy5.5-GC-CA NPs. Cy5.5-GC-CA NPs contained up to 6.28 wt% gadolinium and successfully visualized tumor in the T1-weighted MR image as well as the NIRF image. In addition, these NPs displayed long-term and stable blood circulation and high tumor selectivity due to the enhanced permeability and retention (EPR) effect of tumor tissues. This optical/MR dual imaging probe offered an excellent spatial resolution of tumor tissues with high sensitivity, presenting a promising way for cancer imaging. Nevertheless, gadolinium-based contrast agents can trigger the development of nephrogenic systemic fibrosis, a fibrosing disorder found in patients with renal impairment. Thus, superparamagnetic iron oxide NPs (SPIOs) were utilized as an alternative to gadolinium [13]. The NIRF and MRI dual-modality imaging system using GC, Cy5.5, and SPIOs was effective in displaying tumor regions in T2-weighted images with high tumor specificity. These results demonstrated the feasibility of using GC NP-based optical/MR dual imaging for the early cancer detection. Besides optical/MR dual-modality imaging, GC NPs might also be used for various multimodal diagnostic imaging such as optical/CT, optical/US, and optical/PET, and even for trimodal imaging to increase the opportunities for the successful early diagnosis of tumors. 

## 3. GC Derivatives for Drug Delivery 

### 3.1. GC Derivatives for the Delivery of Antimicrobial Agents 

**Figure 3:**
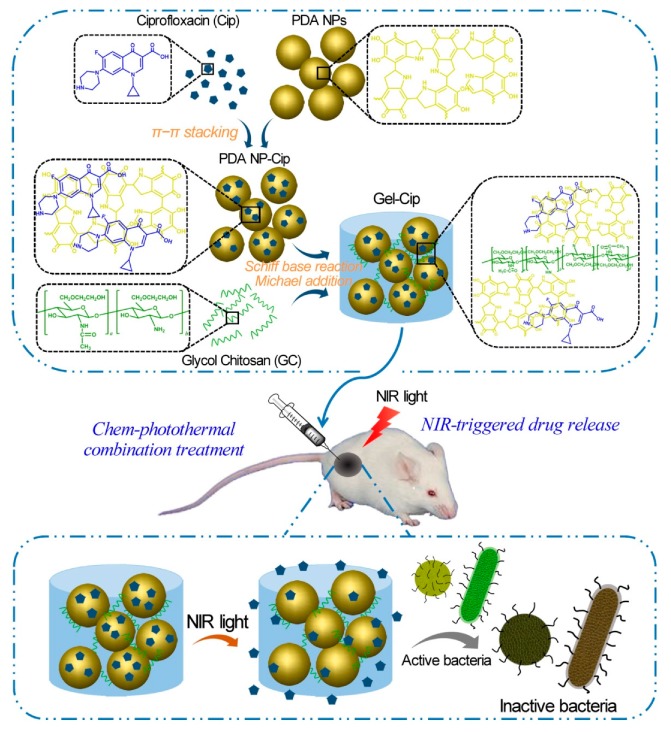
Schematic of the synthetic strategy and bacterial inactivation of Gel-Cip. Reproduced with permission from Ref. [23]. Copyright 2018 Elsevier Ltd.

GC is water-soluble, biocompatible, and biodegradable, serving as a good carrier to deliver antimicrobial agents in active forms for the applications in wound dressing, tissue adhesive, and hemostasis [17]. GC-derived NPs [18,19,20], microspheres [21], and hydrogels [22,23] have been utilized to carry various antibiotics like colistin [22], chlorhexidine acetate [21], and ciprofloxacin [23], silver ions [20], and photothermal therapy (PTT) agents [18,19,23]. Recently, several studies have reported the applications of GC derivatives for antimicrobial PTT using NIR light [18,19,23]. For instance, a pH-sensitive NP system consisting of polyaniline-conjugated GC (PANI-GCS) was developed [18]. This system specifically gathered pathogenic bacteria in the acidic abscesses and destroyed them photothermally by NIR light. Hydrophobic PANI, a PTT agent, was covalently conjugated with GC via its highly reactive amine groups to generate an amphiphilic polymer (PANI-GCS) that self-assembled into NPs in an aqueous solution. Under normal physiological conditions, the PANI-GCS NPs had neutral surface charges and poorly bound to the neighboring host cells, but became positively charged at the sites of acidity-associated focal infections and strongly interacted with the negatively-charged bacterial cell walls via electrostatic interaction. Thus, the bacteria were aggregated in situ and eradicated under the irradiation of NIR light through the PANI-induced PTT. Meanwhile, this approach reduced the damage to tissues and accelerated wound healing by lowering the ambient tissue temperature. Similarly, another PTT compound carboxyl graphene was grafted with GC to realize an enhanced photothermal treatment of focal infections [19]. In a recent work, our group has fabricated a thermo-sensitive hydrogel by mixing ciprofloxacin (Cip, a potent antibiotic)-loaded polydopamine (PDA) NPs and GC, and the resultant hydrogel (termed Gel-Cip) was injectable and could be used for on-demand antibiotic release to combat bacterial infection (Figure 3) [23]. Bacteria were trapped on the surface of Gel-Cip due to the positive charges of GC and the adsorbability of PDA NPs, and killed synergistically by the Cip released from Gel-Cip and local hyperthermia generated by PDA NPs upon NIR light irradiation. Furthermore, Gel-Cip exhibited an outstanding wound healing ability in the *Staphylococcus aureus*-infected mouse skin defect model. This hydrogel-based light-activatable system allows for precise antibiotic release, effective bacterium inactivation, and persistent inhibition of bacteria-induced infections. 

### 3.2. GC Derivatives for Anticancer Drug Delivery

Hydrophilic GC is usually conjugated with hydrophobic molecules through various chemical reactions to form amphiphilic GC derivatives, also termed hydrophobically modified GC. Generally, these derivatives can self-assemble in aqueous solutions into NPs that contain inner hydrophobic cores and outer hydrophilic shells. The resultant GC-based NPs can easily incorporate various types of drugs (e.g., hydrophobic anticancer drugs, peptides, and nucleic acids) by hydrophobic/electrostatic interactions or chemical conjugation, and have been intensively employed for cancer theranostics over the past two decades. Recently, hydrogels formed by GC have also attracted increasing attention in drug delivery applications. As shown below, we will discuss the use of GC and its derivatives for delivering anticancer drugs including chemotherapeutics, nucleic acids, and photosensitizers (PSs).

#### 3.2.1. Chemotherapeutics 

Since nitrogen mustard was successfully applied to noticeably eradicate the lymphatic tumor in 1943, chemotherapy has been gradually developed as the first-line therapeutic strategy for cancer treatments with the discovery of diverse cytotoxic and cytostatic compounds [24,25]. Nevertheless, traditional chemotherapeutic drugs have some inherent disadvantages such as poor water solubility, low stability, low tumor-targeting efficiency, and high systemic toxicity [26]. Even worse, they may cause the development of multidrug resistance in cancer cells. Therefore, it is urgent to overcome these limitations of chemodrugs to improve their therapeutic outcomes. GC NPs are a class of promising drug carriers for hydrophobic chemodrugs. Their hydrophobic cores are suitable for encapsulating hydrophobic drugs, while the hydrophilic outer shells can ensure the stability of the nanoagents during blood circulation until they arrive at the target site. A variety of GC NPs including *N*-acetyl histidine (NAcHis)-GC NPs [27], hydrotropic oligomer-conjugated GC NPs [28,29], and GC-5β-CA NPs [30,31,32] have been developed for delivering hydrophobic chemodrugs like paclitaxel [28,29,30,33,34], doxorubicin (DOX) [27,35,36,37], camptothecin [31,38], docetaxel [32], and cisplatin [39]. The benefits of using GC derivatives as chemotherapeutic drug carriers include: (1) protecting the hydrolysis-labile drugs from hydrolysis, (2) solubilizing extremely insoluble drugs, (3) increasing the circulation time of drugs, (4) improving drug loading efficiency, and (5) realizing the tumor-targeted delivery and release of chemodrugs through the tumor-homing ability of GC NPs. Therefore, compared with free chemotherapeutics, the delivery efficiency and therapeutic efficacy of the chemotherapeutics encapsulated in GC can be improved with reduced side effects. The physicochemical and biological properties of GC NPs, such as size, surface charge, colloidal stability, deformability, cytotoxicity, biodistribution, and tumor-targeting ability can be influenced by the loaded anticancer drugs. Therefore, each GC NP-containing formulation that will be loaded with a particular drug should be carefully evaluated and optimized to achieve the best therapeutic outcome. For example, GC-5β-CA NPs have longer circulation time and stronger liver tumor-targeting ability than polystyrene NPs and liposomes with similar diameters [40]. Hydrotropic oligomer-conjugated GC NPs can encapsulate a large amount of paclitaxel, showing longer blood circulation and better therapeutic effectiveness than that of a commercial anticancer nanoagent, Abraxane^®^ [29].

#### 3.2.2. Nucleic Acids 

GC derivatives can be utilized for nucleic acid delivery in addition to the small-molecule chemodrugs. Gene therapy is a promising strategy to treat different diseases, for example, cancer, cystic fibrosis, adenosine deaminase deficiency, and Alzheimer’s disease [41]. Therapeutic nucleic acids include small interfering RNA (siRNA), plasmids, antisense oligonucleotides, aptamers, DNAzymes, ribozymes, small hairpin RNA (shRNA), and microRNA, etc. These therapeutic nucleic acids can change gene expression at the transcriptional or post-transcriptional level, presenting powerful therapeutic effectiveness for treating cancers. However, nucleic acids are negatively charged and not easily internalized by cells with negatively charged plasma membranes. Even worse, they are highly susceptible to enzymatic degradation and easily degraded by endonucleases in serum and the extracellular matrix. Therefore, developing effective gene delivery vectors is important to achieve successful gene therapy. Polymers and cationic lipids have been widely utilized for the effective gene delivery. Among them, GC derivatives are attractive materials as gene carriers, because they possess abundant primary amine groups on their backbones and can interact with the anionic phosphate groups of genes to form complexes. 

To date, GC derivatives have been successfully employed for delivering siRNA [42,43,44,45,46,47] and plasmids [48,49,50,51,52,53] for treating different diseases (Table 1). Nevertheless, the low charge density of GC displayed weak electrostatic interaction with negatively charged nucleic acids, resulting in loosely bound GC/gene complexes with low stability. To solve this problem, GC-5β-CA and PEI-5β-CA were first combined together at a 1:1 weight ratio to generate GC-PEI NPs with increased positive charges [42]. Then, GC-PEI NPs were mixed with RFP-siRNA at a weight ratio of 1:5 to yield condensed and stable siRNA-encapsulated NPs (siRNA-GC-PEI NPs) with nearly 100% loading efficiency. The siRNA-GC-PEI NPs effectively entered the cell cytoplasm and exerted marked gene silencing effect. Meanwhile, the siRNA-GC-PEI NPs prevented RFP-siRNA from degradation in the presence of RNase A. In other studies, thiolated GC (tGC) was used to yield stable NPs with poly-siRNA by both electrostatic interaction and chemical crosslinking [43,44,45,46,47]. These NPs with tumor-targeting abilities were quickly taken up by tumor cells in vivo to inhibit the expression of the gene encoding the vascular endothelial growth factor (VEGF), leading to reduced angiogenesis in tumor tissues. Collectively, the modified GC derivatives are suitable vectors for delivering siRNA, though more efforts should be put to optimize the treatment protocols and deliver genes safely and efficiently for better clinical applications.

#### 3.2.3. PSs

**Figure 4:**
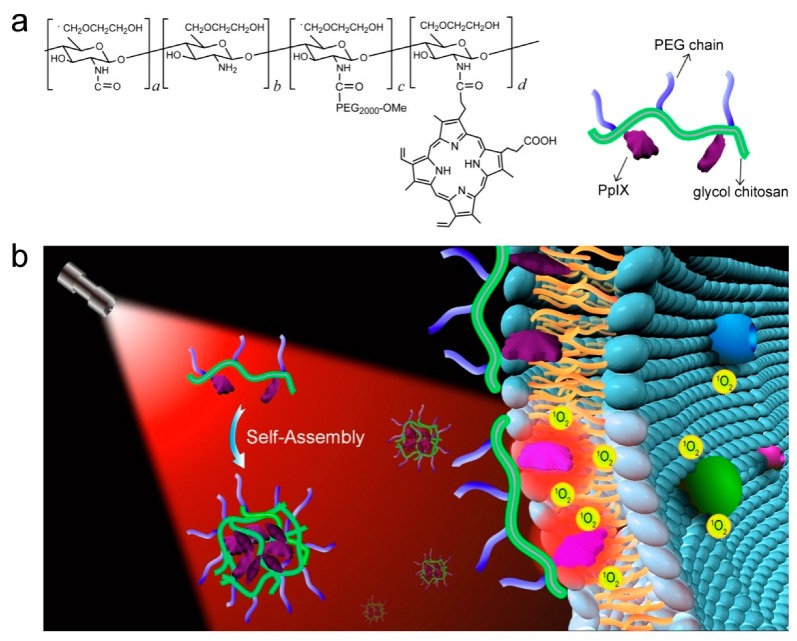
(**a**) Chemical structure of GC-PEG-PpIX and (**b**) its proposed membrane-activatable mechanism for imaging-guided PDT against cancer. Reproduced with permission from Ref. [60]. Copyright 2017 Elsevier Ltd.

**Figure 5:**
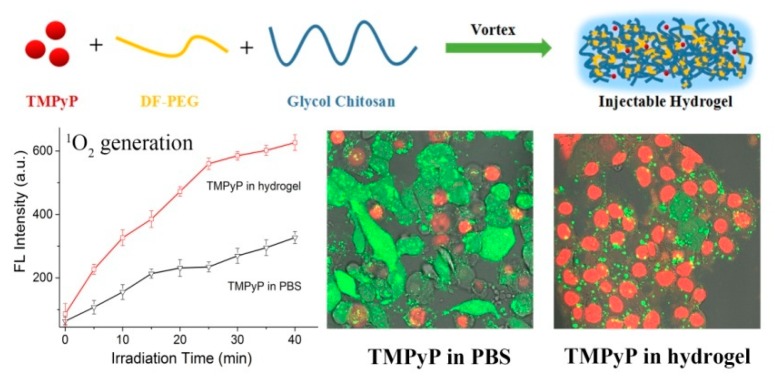
Schematic showing the synthetic route of the TMPyP-loaded hydrogel that exhibits enhanced singlet oxygen generation and improved in vitro PDT efficiency. Reproduced with permission from Ref. [67]. Copyright 2017 American Chemical Society.

Photodynamic therapy (PDT) is considered as a promising noninvasive method to combat cancers with precise spatiotemporal control and reduced side effects. In PDT, the administrated PSs absorb light and convert energy to surrounding molecular oxygen to generate reactive oxygen species (ROS), primarily singlet oxygen, causing cell apoptosis/necrosis [54,55]. PDT is supposed to solve some significant problems in cancer therapy, such as undesirable damage to normal tissues/cells, the drug resistance of chemotherapeutics, and uncontrollable drug delivery. Furthermore, PSs generally possess unique luminescent characteristics to monitor themselves in tumor tissues, acting as theranostic agents for imaging-guided cancer therapy [56]. Nevertheless, most currently available PSs are hydrophobic and have poor water solubility, which prevents them from targeting and entering cells and thus severely compromises their PDT effects [57]. GC derivatives can be utilized to load hydrophobic PSs, including protoporphyrin IX (PpIX) [58,59,60], chlorin e6 (Ce6) [61,62,63], and fullerene [64,65,66], via physical encapsulation or chemical conjugation to enhance their solubility and dispersion in aqueous solutions. The physical encapsulation of hydrophobic PSs into GC NPs has been demonstrated to be able to realize efficient PDT both in vitro and in vivo, but the PS-loaded GC NPs are usually unstable and suffer from burst drug release during circulation in vivo [58,61], leading to poor delivery efficiency, low therapeutic effect at tumor sites, and undesired damage to normal tissues. To avoid these limitations, PSs can be chemically coupled with GC NPs [59,60,61,62,63,64,65,66]. The PS-conjugated GC NPs present a prolonged circulation time and accumulate more specifically in tumor issues, showing better therapeutic efficacy and lower photo-cytotoxicity than PS-loaded GC NPs [59,60,61]. For instance, protoporphyrin IX (PpIX) and polyethylene glycol (PEG) were chemically conjugated with GC to yield GC-PEG-PpIX NPs in aqueous solutions (Figure 4) [60]. The fluorescence of PpIX units in the inner core of the GC-PEG-PpIX NPs was highly quenched owing to the strong *π*–*π* stacking between the neighbouring PpIX molecules; however, when encountering plasma membranes, the GC-PEG-PpIX NPs disassembled and stably attached to the plasma membranes through the hydrophobic anchoring of the PpIX moieties (Figure 4), which effectively relieved the self-quenching of PpIX, resulting in significantly enhanced fluorescence and singlet oxygen (^1^O_2_) production upon laser irradiation. The resultant ^1^O_2_ damaged the plasma membranes, allowing more cellular uptake of GC-PEG-PpIX NPs to promote cell death upon further laser irradiation. In addition, GC-PEG-PpIX NPs showed negligible systemic toxicity and good hemocompatibility, which is beneficial for its future clinical applications. Except hydrophobic PSs, the water-soluble PSs like meso-tetrakis(1-methylpyridinium-4-yl)porphyrin (TMPyP) can be encapsulated into an injectable hydrogel generated by GC and dibenzaldehyde-terminated telechelic poly(ethylene glycol) (Figure 5) [67]. The TMPyP-loaded GC hydrogel displayed higher ^1^O_2_ generation, much longer tumor retention, and enhanced fluorescence intensity than free TMPyP, leading to robust imaging-guided PDT against cancer.

**Figure 6:**
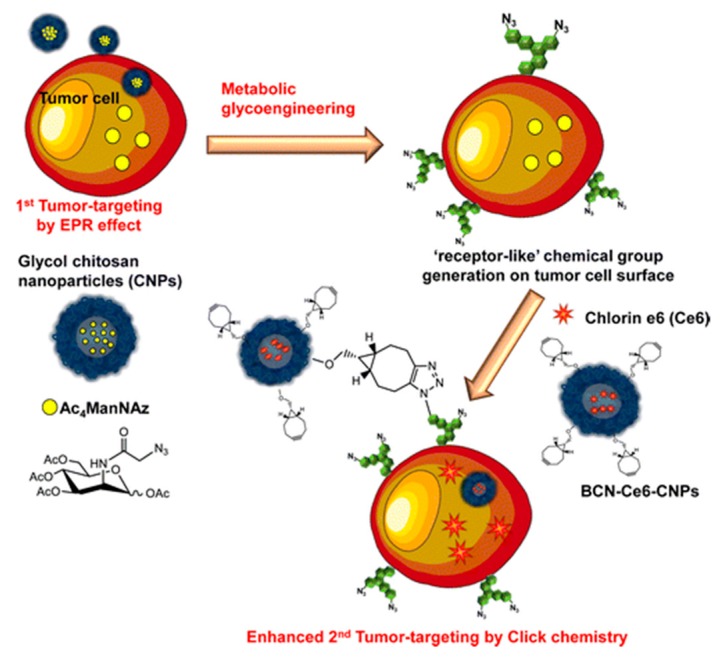
Schematic demonstration of the two-step in vivo tumor-targeting strategy for delivering photosensitizer Ce6 through metabolic glycoengineering and click chemistry. Reproduced with permission from Ref. [62]. Copyright 2014 American Chemical Society.

To deliver PSs to tumor cells specifically and effectively, a novel two-step PDT strategy based on metabolic glycoengineering and click chemistry was developed [62]. Tetraacetylated *N*-azidoacetyl-d-mannosamine (Ac_4_ManNAz), the precursor for azide group generation, was loaded into the amphiphilic glycol chitosan-5β-cholanic acid NPs (CNP) through hydrophobic interaction, yielding Ac_4_ManNAz-CNP (Figure 6). On the other hand, bicycle [6.1.0]nonyne *N*-hydroxysuccinimide ester II (BCN-NHS) and Ce6 were conjugated with CNP to obtain BCN-Ce6-CNP. Intravenous injection of Ac_4_ManNAz-CNP produced azide groups on the cell surface of tumor tissues by site-specific metabolic glycoengineering, which could enhance the tumor-targeting ability of BCN-Ce6-CNP injected intravenously by copper-free click chemistry in vivo. As expected, substantial BCN-Ce6-CNP were selectively delivered to tumor cells, achieving an improved cancer therapeutic outcome in animal studies. Stimuli-responsive GC derivatives have also been developed for delivering and releasing PSs to tumor cells with superb specificity triggered by pH [63,66] or glutathione (GSH) [68]. Considering that solid tumors have a lower pH and higher GSH level than normal tissues, pH-sensitive or redox-responsive GC derivatives are promising for targeted cancer PDT without damaging the normal tissues. 

## 4. GC-Based Multifunctional Platforms for Advanced Therapeutics

As we summarized above, GC derivatives have been widely applied as effective drug delivery carriers due to their unique physiochemical and biological properties. Thanks to its ease of chemical modification and capability to encapsulate various molecules, GC represents an ideal polymeric carrier to construct drug delivery systems with many functions for advanced therapeutics towards cancer. For example, targeting ligands, stimuli-sensitive moieties, and imaging agents can be conjugated with GC molecules for imaging-guided targeted drug delivery and controlled drug release. A variety of anticancer chemodrugs and photoresponsive agents can be loaded into the GC-based NPs for effective and combination cancer therapy. We summarize the recent progress on this part as below.

### 4.1. Tumor-Targeted Drug Delivery

The use of free drugs for anticancer applications typically suffers from indiscriminate distribution of the drugs in vivo due to the lack of tumor selectivity, which will compromise their therapeutic efficacy and cause systemic toxicity [69]. NPs as drug carriers can sufficiently target and accumulate in tumor tissues through the EPR effect, because of the hyper vascularization, leaky vascular architecture, and poor lymphatic drainage of tumors [70]. GC NPs have been well-known for their passive tumor-homing ability through EPR effect and have been used to deliver chemodrugs, genes, and PSs specifically to tumor tissues [30,42,43,47,59,71]; however, the tumor-homing efficiency of GC NPs is generally affected by their molecular weight [5] and the tumor microenvironment [72]. To some extent, the use of higher molecular weight GC endows the GC NPs with longer blood circulation and better tumor accumulation [5]. Furthermore, tumors with abundant and aberrant neovascularization and low extracellular matrix contents are more suitable for the accumulation of GC NPs [72]. All these factors should be taken into account when designing GC NPs for tumor-targeted drug delivery. Nevertheless, the passive tumor-targeting efficiency of NPs is usually unsatisfactory in poorly developed vasculature regions, and thus active targeting strategies using targeting ligands are needed.

Targeting ligands can specifically recognize and bind to their corresponding receptors expressed on tumor cells. Conjugation of these ligands to GC-based delivery systems enables active tumor-oriented delivery with enhanced therapeutic efficacies and reduced adverse side effects. To this end, transferrin [73], RGD peptide [74], interleukin 4-receptor (IL-4R) peptide [75], and folic acid [62], were reported to be chemically conjugated to the GC backbone to interact with transferrin receptors, integrin α_v_β_3_, IL-4R, and folate receptors, respectively. For example, hydrophobically modified glycol chitosan (HGC) was reacted with SMCC (4-(*N*-maleimidomethyl)cyclohexane carboxylic acid *N*-hydroxysuccinimide ester) to provide the maleimide group for the conjugation with the thiol group of IL-4R peptide, resulting in the peptide-tagged HGC NPs (HGC-I4R) [75]. HGC-I4R was loaded with paclitaxel (PTX) to obtain PTX-HGC-I4R NPs. PTX-HGC-I4R NPs selectively targeted human lung squamous carcinoma (H226) cells overexpressing IL-4R, noticeably inhibiting H226 tumor growth. Except the above-mentioned ligands, other ligands like aptamer, antibody, galactose, hyaluronic acid, and glycyrrhizin might also be coupled with GC derivatives to achieve efficient tumor-targeted delivery [76]. However, these ligand-based active tumor-targeting strategies can be severely affected by the inter- and intra-tumor heterogeneity [77]. To circumvent the limitation, an artificial active targeting approach was developed utilizing metabolic glycoengineering and bioorthogonal click chemistry [62,78]. Taken together, GC-based delivery systems can be designed variably according to the complexity and heterogeneity characteristics of tumors for optimizing the efficiency of tumor-targeted delivery. 

### 4.2. Stimuli-Responsive Drug Release 

Despite the promising results achieved in the laboratories and preclinical animal models, the translation of nanocarriers from the laboratory to the bedside is largely hampered by the premature drug release during blood circulation and unwanted accumulation in normal tissues [79]. Aiming at these drawbacks, stimuli-sensitive materials were integrated into nanocarriers to trigger the release of drugs precisely in tumor issues and cells, in response to endogenous stimuli like pH, redox potential, enzymes, reactive oxygen species, and hypoxia, or exogenous ones such as light, US, temperature, and magnetic field [79]. The primary amine groups of the GC backbone can be modified with different stimuli-responsive moieties that are sensitive to pH [38,80,81,82,83,84], glutathione (GSH) [36,53,68], US [85], light [38,63], and hypoxia [86].

In tumors, extracellular pH ranges from 6.0 to 7.2 [87] and the intracellular pH is 5.0–6.0 in endosomes and 4.0–5.0 in lysosomes [88], which are lower than pH 7.4 in the blood and normal tissues and can be employed for constructing tumor-specific drug release systems. Generally, pH-responsive GC NPs can be developed using acid-labile bonds like imine bonds [38], *N*-*cis*-aconityl linker [80,81], and ester bonds [82], or pH-responsive moieties such as 3-diethylaminopropyl isothiocyanate [83] and poly(2-(diisopropylamino)ethyl methacrylate) (PDPA) [84]. Park et al. chemically conjugated GC with DOX using an acid-labile *N*-*cis*-aconityl linker, yielding GC-ADR that self-assembled into NPs with a size distribution of 204–238 nm in aqueous solutions [80]. The amount of DOX released from GC-ADR under pH 4 was 4 times higher than that under pH 7. In another study, Yang et al. fabricated estrone-modified GC NPs (GCNP-ES) using a pH-responsive moiety PDPA for achieving efficient PTX delivery in breast cancer cells [84]. After cellular internalization, GCNP-ES disassembled under acid conditions and efficiently released PTX in tumor cells. 

GSH is one of the most important reductants in mammalian cells with extremely low concentrations of 2–20 µM in the blood or extracellular compartments [89], but its concentration increases to 0.5–10 mM in both the intracellular environment and tumor tissues. This increased GSH concentration can be utilized to realize redox-sensitive tumor-specific drug delivery. To this end, GC was grafted with the PS pheophorbide a (PheoA) [68] or PEI [53] via reducible disulfide bonds, obtaining conjugates PheoA-ss-GC and GCS-ss-PEI, respectively. Disulfide bonds are stable at low levels of GSH but can be rapidly cleaved by GSH at high concentrations in tumor cells. PheoA-ss-GC conjugates self-assembled into core–shell structured NPs (PheoA-ss-CNPs) in aqueous condition. Notable photoactivity of PheoA-ss-CNPs was found in the intracellular reductive environment of tumor cells via the cleavage of the disulfide bonds, remarkably blocking tumor growth [68]. Except for the utilization of reducible disulfide bonds, GC was also integrated with lipoic acid (LA) to generate GSH-responsive core-crosslinked nanocarriers GC-LA for intracellular DOX delivery into A549 cancer cells [36]. 

US can penetrate tissues deeply and realize tumor-specific drug release spatiotemporally and noninvasively, improving therapeutic efficiency and preventing side effects. The echogenic NPs with US contrast agents produce bubbles in target tissues, generating cavitation effect that enhances the penetration and the cellular uptake of drugs in target tissues. US-responsive and tumor-homing echogenic GC NPs (Echo-CNPs) were constructed by encapsulating DOX and bioinert perfluoropentane (PFP), a US contrast agent, in CNPs [85]. Echo-CNPs exhibited the prolonged echogenicity via the phase-transition from liquid to microbubbles at the body temperature as well as excellent tumor-targeting due to the EPR effect. Once arriving at tumor tissues, DOX was released from Echo-CNPs upon external US irradiation, achieving simultaneous cancer-oriented US imaging and US-responsive delivery for anticancer drugs.

**Figure 7:**
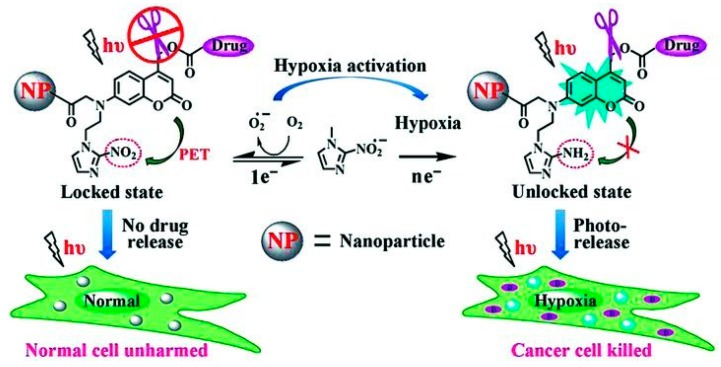
The hypoxia-responsive phototrigger for tumor-specific drug release. Reproduced with permission from Ref. [86]. Copyright 2013 Wiley.

A number of stimuli-responsive delivery systems derived from GC have been developed by combining endogenous and exogenous stimuli, such as pH/light [38,63,66] and hypoxia/light [86], for precise tumor treatment. The rapid tumor growth is accompanied with increased oxygen consumption, leading to hypoxia in tumor tissues. For instance, The tumor-hypoxia activated phototrigger (TAP) complex was fabricated by conjugating GC with a prepared complex consisting of etoposide as the anticancer drug, nitroimidazole as the hypoxia sensor, and 7-aminocoumarin as the photo-sensitive agent (Figure 7) [86]. In TAP, etoposide was caged using 7-aminocoumarin that was further locked by nitroimidazole under normoxia condition. Only the intracellular hypoxia effectively induced the irreversible bioreduction of nitroimidazole, enabling the photocleavage between eoposide and 7-aminocoumarin and the release of free etoposide to kill cancer cells upon light irradiation. This approach provides highly selective photorelease of anticancer drugs to hypoxic tumor cells, but not to healthy oxygenated cells. In summary, modification of GC derivatives with stimuli-responsive moieties allows for the release of anticancer drugs specifically at tumors mediated by endogenous and exogenous triggers for improved delivery and therapeutic efficiencies. 

### 4.3. Cancer Theranostics 

The advances in nanotechnology have led to the development of novel multifunctional nanomaterials that entail the simultaneous delivery of imaging agents and therapeutic drugs for cancer imaging and therapy, which is termed “cancer theranostics”. Effective theranostic systems that possess both diagnostic functions and therapeutic effects have attracted extensive attention due to their excellent properties, including real-time monitoring capability, high efficiency, and the ease of operation. In recent years, GC-based materials have been developed as dual-purpose theranostic systems for simultaneous diagnosis and therapy. They have shown great potential in different cancer therapies like chemotherapy [30,85], gene therapy [43], PDT [59,60,67], and PTT [90]. For example, Cy5.5-labeled GC-5β-CA NPs were developed by chemically conjugating Cy5.5 to GC-5β-CA conjugates [5]. They showed high stability, superior tumor-targeting ability, and strong fluorescence in vivo. These NPs were loaded with PTX, yielding novel theranostic NPs (i.e., PTX-CNPs) [30]. PTX-CNPs were highly useful for early detection of cancer and monitoring drug delivery in vivo. They exhibited significantly increased tumor-homing ability with low nonspecific uptake by other tissues in SCC7 tumor-bearing mice. In this way, the biodistribution and tumor-targeting ability of GC-derived systems can be well monitored by measuring their in vivo fluorescence. Meanwhile, PS-containing GC delivery systems can generally realize imaging-guided therapy, as PSs are usually fluorescent dyes and serve as good signal emitters themselves [59,60,67]. These GC-based theranostic platforms exhibited excellent tumor-targeting capability, appreciable colloidal stability for long circulation in the bloodstream, good deformability for reduced in vivo filtration by the liver or spleen, or fast cellular uptake. Due to these advantages, GC-derived theranostic systems show effective therapeutic efficacies for cancer treatment, which may play a key role in the future biomedical applications.

### 4.4. Combination Cancer Therapy

**Figure 8:**
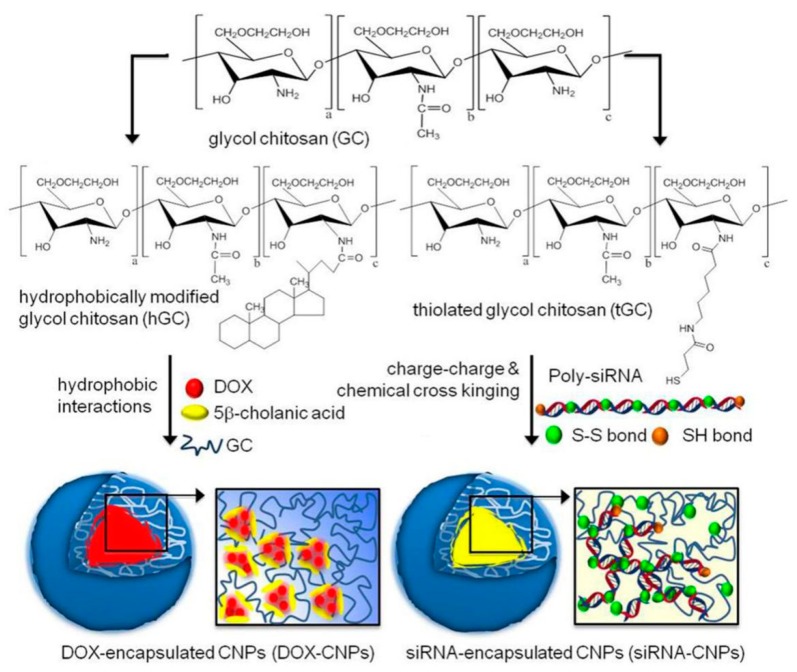
Synthetic routes of DOX-CNPs and siRNA-CNPs. Reproduced with permission from Ref. [91]. Copyright 2014 Nature Publishing Group.

**Figure 9:**
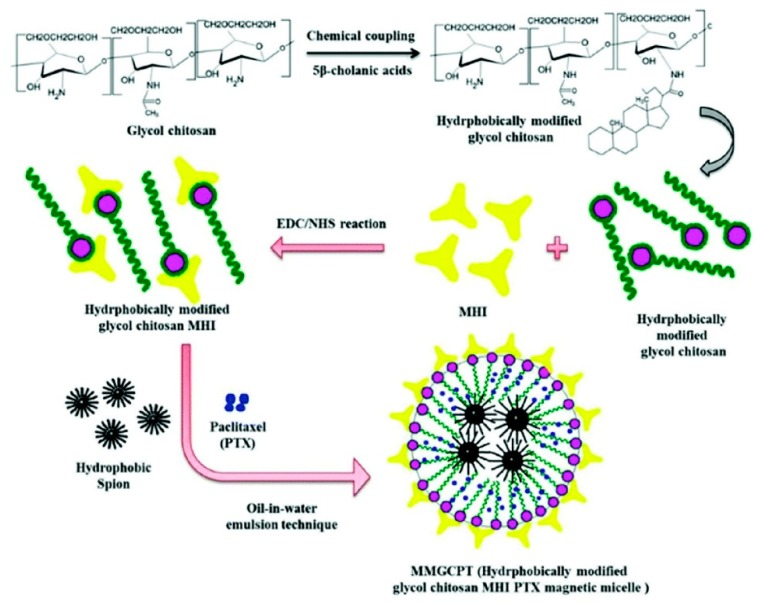
Synthetic route of the MMGCPT nanocarrier. Reproduced with permission from Ref. [90]. Copyright 2018 Royal Society of Chemistry.

Even though chemotherapy has advanced greatly in the past decades, it still has some limitations to overcome, such as severe side effects, drug resistance, and high relapse rates. Therefore, combination therapy by integrating two or more treatment strategies with orthogonal mechanisms and synergistic therapeutic effects is highly desirable. GC NPs serve as an effective platform that can carry multiple therapeutic compounds to achieve maximal efficacy and overcome drug resistance [90,91]. For instance, DOX and Bcl-2 siRNA were loaded in GC NPs separately, generating DOX-CNPs and siRNA-CNPs, respectively (Figure 8) [91]. DOX-CNPs and siRNA-CNPs were delivered to tumor sequentially, successfully suppressing the up-regulation of Bcl-2 caused by the repeated treatment of DOX-CNPs. The sequential treatment showed higher cytotoxic effect than single treatment of DOX-CNPs on acquired chemotherapeutic resistant PC3 tumor cells. In another study, Sasikala et al. integrated MRI/optical dual-modal imaging and combined chemo-photothermal therapy into one nanosystem [90]. HGC was chemically bound with a heptamethine cyanine dye MHI-148 for PTT and NIRF imaging through the 1-ethyl-3-(3-dimethylaminopropyl) carbodiimide (EDC)/*N*-hydroxysuccinimide (NHS) coupling reaction, which was then co-loaded with PTX, a chemotherapeutic agent, and hydrophobically-modified superparamagnetic iron oxide NPs (SPIONs), a PTT and MRI agent, to form the hydrophobically-modified glycol chitosan MHI PTX magnetic micelle (termed MMGCPT) (Figure 9) [90]. MMGCPT displayed satisfactory in vivo performance, including the highly sensitive tumor imaging and efficient tumor eradication, which outperformed the therapeutic efficiency of monotherapies.

## 5. Conclusions and Outlook

As summarized in this review, GC derivatives have been successfully applied to deliver antimicrobial agents and anticancer drugs such as chemodrugs, genes, and PSs, either by physical encapsulation or chemical conjugation. GC can be modified with hydrophobic molecules like 5β-CA [30,31,32], *N*-acetyl-histidine [27], sulfo-LC-SPDP [43], glutathione [92,93], *N*-acetylcysteine [92,93], 2-(4-(vinylbenzyloxy)-*N*,*N*-diethylnicotinamide) [28,29], palmitic acid [94], and LA [36] to obtain amphiphilic GC derivatives that generally can self-assemble into NPs in aqueous conditions. Meanwhile, GC can be directly linked with hydrophobic drugs to generate amphiphilic compounds that can also form NPs for cell imaging and drug delivery. Moreover, GC-derived hydrogels have also been widely used in recent years, benefiting from their excellent drug loading and delivery properties. Consequently, GC-based materials are promising to serve as a highly versatile platform that can be rationally designed for diverse therapeutic purposes, including the tumor-targeted drug delivery via passive targeting based on EPR and/or ligand-based active targeting, on-demand drug release in response to specific stimuli, cancer theranostics, and combination therapy, which are desirably needed for precise and personalized cancer treatments in the clinic. 

The use of GC derivatives for cell imaging and drug delivery has several advantages, including superb tumor-homing ability in the case of GC NPs based on EPR effect, low cytotoxicity, ease of chemical modification, great biocompatibility, and biodegradability. Nevertheless, some challenges still exist currently when using GC derivatives, which deserve continuous efforts in material engineering, biology, and medical science. First, the biocompatibility of GC derivatives in some studies are taken for granted by stating that chitosan has been approved by the United States Food and Drug Administration (FDA) as a wound dressing material. However, the biocompatibility of GC derivatives can be influenced by different factors such as structure, formulation, and application condition, which requires careful safety evaluations. Second, the complexity and heterogeneity of pathologic tissues significantly influence the ultimate delivery efficiency and therapeutic outcomes of GC-based carriers. For instance, the tumor accumulation of GC NPs may rely heavily on the tumor types, mainly due to the different tumor microenvironments. Therefore, the use of GC molecules for cancer theranostics should be evaluated in a case-by-case manner. Third, the use of GC derivatives for imaging and drug delivery applications is still in its infancy, and still require deep investigations and comprehensive biosafety evaluations to prompt the clinical translation of GC derivatives as the carriers of antimicrobial and anticancer drugs. Fourth, the current synthetic strategies of GC derivatives are not simple and well-defined enough to realize large-scale production with controllable quality for commercialization, which is important for clinical translation. Finally, to the best of our knowledge, the use of GC derivatives for carrying radioactive agents for radiotherapy, another conventional and popular cancer treatment modality in the clinic, has not been reported. Future efforts must be put on the development of innovative GC-based strategies to overcome these challenges.

Besides being used as drug carriers, GC derivatives have also found wide applications in other biomedical fields. As an example, a thermo-sensitive hydrogel fabricated by an *N*-hexanoyl GC derivative was used to coat the surfaces of cell culture dishes for in vitro three-dimensional cell culture [95]. In another example, a photocrosslinkable hydrogel system was prepared using methacrylated GC, montmorillonite, and riboflavin for bone tissue engineering [96]. Nevertheless, the potential applications of GC derivatives are yet to be explored for template synthesis of biomaterials, tissue engineering, and treatments of different diseases like cardiovascular disease, inflammation, Alzheimer’s disease, and microbial infections. In summary, the versatility of GC has inspired researchers to produce various GC-based drug carriers or imaging agents and successfully apply them to the treatment of cancers and microbial infections. We believe that the studies on GC derivatives will continue to contribute to the future biomedical applications.

## Figures and Tables

**Table 1:** GC derivatives for gene delivery.

GC	Gene	Synthetic Method	Form	Size (nm)	Application	Ref.
GC-5β-CA	siRNA	Electrostatic interaction	NPs	350	Inhibition of red fluorescent protein (RFP) expression in tumor-bearing mice	42
tGC	Poly-siRNA	Electrostatic interaction and chemical crosslinking	NPs	300	Knockdown of tumor proteins	43
tGC	Dual-poly-siRNA	Electrostatic interaction and chemical crosslinking	NPs	243	Dual-gene silencing of VEGF and Bcl-2	44
tGC	Poly-siRNA	Electrostatic interaction and chemical crosslinking	NPs	270	Down-regulation of P-glycoprotein (Pgp) to overcome Pgp-mediated multidrug resistance	45
tGC	Poly-siRNA	Electrostatic interaction and chemical crosslinking	NPs	310	Inhibition of the gene expression of VEGF	46
tGC	Poly-siRNA	Electrostatic interaction and chemical crosslinking	NPs	240	Down-regulation of the expression of VEGF gene in PC-3 cells	47
GC-5β-CA	Plasmid	Hydrophobic interaction	NPs	277	Gene delivery	48
Quaternized chitosan oligomers	Plasmid	Electrostatic interaction	–	–	Gene delivery to epithelial cell lines	49
GMP	Plasmid	Electrostatic interaction	Nanorod	–	Gene delivery	50
GMP	Plasmid	Electrostatic interaction	–	–	Gene delivery to human adipose-derived mesenchymal stem cells	51
GC	Plasmid	Electrostatic interaction	NPs	250	Retinal gene delivery	52
GCS-ss-PEI	Plasmid	Electrostatic interaction	Pseudo-spherical	45	Redox-responsive gene delivery	53

GC-5β-CA: glycol chitosan-5β-cholanic acid; tGC: thiolated glycol chitosan; GMP: glycol chitosan–methyl acrylate-polyethylenimine; GCS-ss-PEI: a glycol chitosan-based disulfide bond-containing polyethylenimine derivative.
